# Identification and Characteristics of Signature Whistles in Wild Bottlenose Dolphins (*Tursiops truncatus*) from Namibia

**DOI:** 10.1371/journal.pone.0106317

**Published:** 2014-09-09

**Authors:** Hannah Joy Kriesell, Simon Harvey Elwen, Aurora Nastasi, Tess Gridley

**Affiliations:** 1 Department of Conservation Biology, Georg-August University Göttingen, Göttingen, Germany; 2 Mammal Research Institute, Department of Zoology and Entomology, University of Pretoria, Hatfield, Gauteng, South Africa; 3 Namibian Dolphin Project, Walvis Bay, Namibia; 4 Sapienza Università di Roma, Dipartimento di Scienze della Terra, Rome, Italy; Università degli Studi di Napoli Federico II, Italy

## Abstract

A signature whistle type is a learned, individually distinctive whistle type in a dolphin's acoustic repertoire that broadcasts the identity of the whistle owner. The acquisition and use of signature whistles indicates complex cognitive functioning that requires wider investigation in wild dolphin populations. Here we identify signature whistle types from a population of approximately 100 wild common bottlenose dolphins (*Tursiops truncatus*) inhabiting Walvis Bay, and describe signature whistle occurrence, acoustic parameters and temporal production. A catalogue of 43 repeatedly emitted whistle types (REWTs) was generated by analysing 79 hrs of acoustic recordings. From this, 28 signature whistle types were identified using a method based on the temporal patterns in whistle sequences. A visual classification task conducted by 5 naïve judges showed high levels of agreement in classification of whistles (Fleiss-Kappa statistic, κ = 0.848, *Z* = 55.3, *P*<0.001) and supported our categorisation. Signature whistle structure remained stable over time and location, with most types (82%) recorded in 2 or more years, and 4 identified at Walvis Bay and a second field site approximately 450 km away. Whistle acoustic parameters were consistent with those of signature whistles documented in Sarasota Bay (Florida, USA). We provide evidence of possible two-voice signature whistle production by a common bottlenose dolphin. Although signature whistle types have potential use as a marker for studying individual habitat use, we only identified approximately 28% of those from the Walvis Bay population, despite considerable recording effort. We found that signature whistle type diversity was higher in larger dolphin groups and groups with calves present. This is the first study describing signature whistles in a wild free-ranging *T. truncatus* population inhabiting African waters and it provides a baseline on which more in depth behavioural studies can be based.

## Introduction

Social animals need to keep in contact and contact vocalisations are fundamental to many animal communication systems [1], [2], [3], [4]. Contact calls are particularly necessary in environments where animals are widely dispersed and/or visual communication is difficult. A signature whistle type is a learned, individually distinctive whistle type within a dolphin's repertoire that broadcasts the identity of the whistle owner [5]. Identity information is encoded independently of voice cues in the distinct frequency contour of signature whistles [6] producing a designed individual signature signal [7] - a feature unique to dolphins amongst non-human animals [8]. Bottlenose dolphins (*Tursiops* spp.) use signature whistles as contact calls to maintain group cohesion [9], [10]. They help facilitate reunions between familiar individuals including mother and calf pairs [11] and male alliances [9]. Groups exchange signature whistles before joining [12]. Vocal copying of signature whistles takes place between close associates [13] and may be used to address conspecifics [14]. Such vocal labelling is unusual in the animal kingdom and indicates complex cognitive functioning in bottlenose dolphins.

The signature whistle is learned within the first year of life [15] and whistle development is influenced by auditory experience [5]. Calves model their whistles on the sounds they hear [16], [17] and most develop signature whistles that are different from their closest associates [17], [18]. However, some male calves develop signature whistles that resemble their mothers' signature whistle [18]. Once formed, the frequency modulation pattern remains stable over decades [9], [19], but the signature whistle types of males in alliances can become more similar over time [20], [21], while retaining individual identity information [21].

Despite being studied for 50 years [5] and described in over 300 individuals during this period [5], [8], studies of wild, freely interacting dolphins are still rare. The vast majority of research on wild common bottlenose dolphin (*T. truncatus*) stems from a single study population in Sarasota Bay (Florida, USA) where many individuals' signature whistles have been identified during brief capture-release events [8] enabling researchers to later track these whistles when animals are freely interacting [9], [22]. Only recently has a method been developed to confidently identify signature whistles in freely interacting dolphins based on the temporal production of these calls [23]. Observing that signature whistles of common bottlenose dolphins are often produced in sequences with inter-whistle intervals (see terminology section) from 1 to 10 s, Janik *et al.*, [23] developed a SIGnature IDentification method (SIGID) using a bout analysis approach to identify them in recordings of freely interacting animals [23]. This method has been used to find signature whistles in the repertoires of wild common [12], [14] and Indo-Pacific (*T. aduncus*) [24] bottlenose dolphins, but so far no study has described the acoustic characteristics of signature whistles identified from a wild population using the SIGID method.

Variation between signature whistles can be assessed by examining the frequency modulation pattern of the whistle contour, which differs between individuals (often determined using visual or automated categorisation [25], [26]), or by using standard acoustic parameter measurements, such as start, end, minimum or maximum frequency [27], [28]. The temporal production of signature whistles, for example the inter-whistle or inter-loop intervals may also differ between individuals, populations or recording contexts [29], [30], [31]. Studies working with whole whistle repertoires, i.e. those that do not differentiate between signature and non-signature whistles during analysis, suggest that whistle acoustic parameters and/or production vary with geographic location [27], [32], [33], behavioural context [33], [34], [35], [36] and group composition [37]. However, as individually distinctive contact calls, the features of signature whistles may differ systematically from those of other whistle types in the repertoire of bottlenose dolphins. Since signature whistles make up around 50% of the whistle repertoire in freely interacting animals [22], and are developed through vocal production learning [5] it seems highly likely that geographic variation in signature whistle characteristics exists. If so, basing our understanding of these characteristics on information from just one wild population (Sarasota Bay), as well as captive studies [16], [30], [38], may limit our understanding of these signals.

Here we use SIGID [23] to identify signature whistles from a wild, freely interacting population of common bottlenose dolphins inhabiting the central coastal region of Namibia. We provide a baseline description of signature whistle occurrence, acoustic parameters and temporal production and compare signature whistle characteristics between Walvis Bay, Namibia and Sarasota Bay, Florida, USA.

## Methods

### Data collection

Data were collected during 4 different field seasons between 2009 and 2013 (see Table 1 for summary) in Walvis Bay (22° 57′ S; 14° 30′ E), a 10×10 km north facing bay located along the central coast of Namibia. The coastal marine environment of Walvis Bay is characterized by cool water (13.4 to 17.9°C) and by a soft mud and sand sediment bottom [39]. Bottlenose dolphins from the Walvis Bay population range along the Namibian coastline from Lüderitz to at least Cape Cross (approximately 550 km distance) inhabiting the near-shore waters less than 30 m deep. However, as the only embayment of significant size along the central Namibian coastline, Walvis Bay represents the core habitat for this population, providing shelter from strong south westerly swells and good resting and foraging opportunities. The Walvis Bay bottlenose dolphin population is small (approximately 100 individuals) and apparently isolated from other bottlenose dolphin populations along the west coast of Africa [40], [41].

**Table 1:** Summary of research vessel, recording device and amount of data analysed over 4 years of research between 2009 and 2013.

Data collection	Research vessel	Recording device	Hours analysed	Number of contours recorded
Mar 2009	8 m ski boat fitted with twin 80hp 4-stroke Honda engines	Edirol UA-25 sound card to PC	10.80	97
Jun-Aug 2011	5.7 m rigid hulled inflatable boat (RHIB) fitted with twin 50hp 2-stroke Mercury engines	Zoom H4n digital recorder	37.20	1641
*Jun-Aug 2012	5.7 m rigid hulled inflatable boat (RHIB) fitted with twin 60hp 4-stroke Yamaha engines	Zoom H4n digital recorder	27.11	1405
Jan & Jun 2013	5.7 m rigid hulled inflatable boat (RHIB) fitted with twin 60hp 4-stroke Yamaha engines	Zoom H4n digital recorder	4.30	627

*Includes one encounter from the second field site, Lüderitz.

Local weather conditions create predictably calm, flat seas in Walvis Bay during the morning with stronger winds in the afternoons (usually Beaufort 4 or higher). Therefore, boat surveys to conduct focal follows [42] of groups were mostly carried out in the mornings when the probability of finding dolphins was the highest. When dolphins were sighted an encounter was begun. Acoustic data were collected together with standard information on the estimated group size, dispersion and number of calves. A concentrated period of photo-identification was also undertaken at the start of each encounter to determine the individuals present. In order to minimize disturbance, no focal follow lasted more than 4 hrs and most lasted between 1 and 2 hrs. Dolphins were approached from the back or the side, carefully adjusting the vessel speed to match that of the dolphin group or turning the engines off to reduce the disturbance of the engine noise on the dolphins' surface and acoustic behaviour. An established dolphin watching industry operates in Walvis Bay and recordings were often made in the presence of one or more tour boats operating with 4-stroke outboard or inboard diesel motors, which were mostly idling or travelling slowly during encounters with dolphins.

Underwater acoustic recordings of dolphin vocalisations were made using the equipment reported in Table 1. Throughout the study we used a single element High-Tec HTI-96-MIN hydrophone, frequency response of 2 Hz to 30 kHz (±1 dB), sensitivity of −170 dB re 1 V/µPa, sampling at 96 kHz. The hydrophone was weighted with a 1 cm diameter steel chain and lowered to 2 to 3 m below the water surface. When dolphins were found in waters <3.5 m deep, the hydrophone depth was reduced accordingly. Acoustic recordings were made whenever possible when the research vessel was stationary, idling or travelling slowly (less than 8 kn). However, in certain encounters recordings could not be made, were interrupted or terminated, for instance when dolphins were positioned in the surf zone, in shallow waters or when groups were engaged in fast travel.

In addition to the recordings made in Walvis Bay, we analysed 2 recordings made during a single encounter from a second field site, Lüderitz (26° 35′ S; 15° 08′ E, approximately 450 km south of Walvis Bay) on the 1^st^ of June 2012, using the collection methods described above. Photo-identification matches of individuals seen during this encounter demonstrated that many of these were bottlenose dolphins from the Walvis Bay population which were encountered during focal follows to collect acoustic data either before or after the encounter in Lüderitz.

### Terminology

To prevent confusion and aid comparisons with other whistle studies, we apply the following terminology throughout. We use the term ‘contour’ to describe the very basic unit of our analysis. A contour is any narrow-band tonal signal lasting 0.1 s or more with at least part of the fundamental frequency above 3 kHz. This distinguishes contours from other narrow-band sounds produced by bottlenose dolphins [43], [44]. Harmonics other than the fundamental were not considered in this analysis and contours interrupted by very short breaks (<0.03 s) were treated as continuous [24], [45]. We use the term ‘whistle’ to describe a unit of 1 continuous contour (either single element or connected multi-loop whistle) or 2 or more repeated contours (loops) separated by a period of silence between 0.03 s and 0.25 s in duration (disconnected multi-loop whistle) [28]. Disconnected multi-loop whistles usually consist of 2 or more loops with the same frequency modulation pattern repeated with a period of silence. Dolphins may also produce signature whistles as 2 or more disconnected loops without a repeated loop structure [28]. To be considered as a whistle unit these loops had to occur in the same sequence produced within 0.03 s to 0.25 s of each other at least 80% of the time [10], [28].

The term ‘whistle type’ is used to describe all whistles of a particular frequency modulation pattern as determined by visual categorization. The term ‘repeatedly emitted whistle type’ (REWT) refers to whistle categories containing whistles that are produced at least twice within a time period of 0.25 s to 10 s during a recording section. For disconnected multi-loop whistles, inter-whistle intervals (IWIs) for REWTs were calculated using the end time of the last loop of a whistle to the start time of the first loop of the next. Using these classifications, ‘signature whistle types’ were identified using the SIGID method [23] (see below), with all signature whistles within a type having the same frequency modulation pattern.

### Identification of whistle types

Visual categorisation of call types is routinely applied in bioacoustic research and can be reliably used to identify signature whistle types [8], [26]. Measurements of inter-observer agreement help to gauge consistency in groupings [46]. The first step in our analysis involved identifying contours and creating a REWT catalogue using visual categorisation to which we could match whistles and use later to identify signature whistles. All acoustic recordings were visually and aurally scanned in the spectrogram display (FFT of 512 and Hanning window) of Adobe Audition (Ver. 2.0 and 4.0) for the occurrence of contours and to identify REWTs. Each contour found was visually assessed and graded based on the signal-to-noise ratio (SNR) (1: signal is faint but visible on the spectrogram, 2: signal is clear and unambiguous, 3: signal is prominent and dominates - see Figure S1 for examples). Every new REWT identified was assigned a unique ID and added to a catalogue containing a selection of the clearest examples of each. Once all recordings had been checked for the occurrence of REWTs, the catalogue was cross-checked by 2 of the authors (HJK and TG) to ensure that each REWT category was unique and mutually exclusive. In one case 2 categories were similar and whistles could plausibly be assigned to either one. Here a conservative approach was adopted and the categories were combined.

Once the catalogue was complete, all acoustic recordings were scanned a second time. Every contour was visually compared to each of the REWT templates in the catalogue. Contours were classified as either a REWT or a variable contour i.e. contours that were not produced in a repeated pattern throughout the entire dataset. In cases where the contour was unclear (i.e. due to masking and/or low SNR), contours were assigned into an ‘unclassified’ bin. This second classification phase was based on frequency modulation pattern alone. Therefore, even if a contour was identified as a single occurrence, it could be classified into a REWT category because on another occasion whistles of the same type had fulfilled the REWT criteria, i.e. were produced at least twice within a time period of 0.25 to 10 s.

At this stage, whistles which were classified into REWTs were assessed according to their loop structure as single element (SE), connected multi-loop (CML) or disconnected multi-loop (DCMLs) whistles. Probable cases of whistle copying [13], [47] were identified as instances where 2 whistles of the same REWT category overlapped in time. Once classification was complete, signature whistle types were identified as REWTs containing 4 or more whistles recorded from the same encounter, where on at least one occasion the whistles were produced in a sequence with 75% or more (i.e. minimum 3 out of 4) occurring within 1 to 10 s. These criteria are based on the SIGID method [23].

### Classification task

To test whether whistle types could reliably be classified into the categories we designated, a visual classification task was set using 5 independent human judges to assess a subset of the data. Ten signature whistle types were chosen from the dataset for the task. For each, 6 whistle repeats were used: 1 to act as a whistle type template and the remaining 5 to be classified by judges. The criteria for including whistles in the task was a good SNR (2 and 3) and that the whistles were not masked; otherwise whistle choice was random. Each whistle was plotted as a spectrogram with standardised time and frequency axis (scales not plotted). Fifty slides were made in Microsoft PowerPoint, each consisting of one of the 50 whistle repeats in the centre surrounded by the 10 signature whistle type templates. Template whistles did not change configuration between slides but the order of slide presentation was randomized for each judge. For the first part of the task, the judges were asked to compare each whistle repeat to all 10 templates and rate the similarity of each on a scale from 1 (the whistle and the template are very different) to 5 (the whistle and the template are very similar). This resulted in a total of 500 pair wise comparisons. The second part of the task was binary and the judges were constrained to assign each whistle repeat to a single ‘most similar’ template category. None of the judges (aged between 26 and 50 years, 3 men and 2 women) had any previous bioacoustic experience. A sixth judge (author HJK) who created the REWT catalogue and classified the REWTs also completed the task. The ratings were compared between the judges using the Fleiss' Kappa statistic [48] to determine inter-observer agreement in call classification and consistency in categorisation (calculated with and without authors' classifications). If judges are in complete agreement in their classification then Fleiss' Kappa statistic (κ) is equal to 1 [49]. If agreement amongst judges is the same as would be expected by chance, then κ is equal to 0.

### Signature whistle acoustic parameters, temporal production and diversity

The frequency parameters of signature whistles were measured from spectrograms in Raven Pro 1.4 [50] software using the selection function (FFT 512, Hanning window, overlap 50%). It was important that only high quality whistles (SNR 2 and 3, not masked, start and end clearly visible) were used in this analysis, as inaccuracy in measurements of call parameters can be introduced if low quality signals are used. For each signature whistle type, we looked for bouts where 4 to 10 high quality whistles were repeated consecutively. We set this lower limit to the minimum number of whistles necessary for a REWT to be defined as a signature whistle type and capped the maximum number to 10 to prevent large variations in sample size of whistle types. Signature whistle types which did not contain at least 4 high quality whistle repeats were discounted from this analysis. The following standard acoustic parameters were measured from each signature whistle: start, end, minimum, maximum and peak frequency, frequency range and whistle duration. The number of inflection points (change from positive to negative aspect or vice versa) was visually assessed together with the aspect of the start and end slopes (rated as 1 for positive, 0 for constant and −1 for negative frequency). Since loops in DCMLs are separated by a period of silence of 0.03 s to 0.25 s in duration, inflection points were calculated separately for each loop and summed for the whistle.

When whistle copying was identified, it was not clear whether the first or second whistle produced was the copy, as copying behaviour is involved in both addressing and matching interactions [13], [14]. As whistle copies can have slightly different acoustic parameters to the original [13], neither whistle from a copying interaction was used in the parameter analysis. Omitting both whistles from the temporal production analysis (details below) would have inflated measurements of IWI. Therefore, for determining temporal production of signature whistles and signature whistle type category sizes, we applied a decision rule. We always assumed the second whistle (overlapping whistle) was a copy and removed this from further analysis whilst retaining the first whistle produced in a copying sequence.

Whistle temporal production was assessed for all signature whistle types, whereby the IWIs of bouts containing 4 or more whistles were measured. We included low SNR whistles (quality 1) in this analysis if they were confidently assigned to a signature whistle type and clearly part of a sequence. Even though they were low SNR, it was important to use these whistles to prevent over-estimation of the IWI measurements. As engine noise (from the research vessel or tour boats) could mask whistle production, sections of high boat noise were identified and IWIs calculated only for bouts occurring at times of moderate, low or no ambient boat noise. As the number of disconnected multi-loop signature whistles was low, loop temporal production (inter-loop interval, ILI) was analysed for all disconnected multi-loops identified.

Individually specific contact calls are often used in situations of group or individual separation as well as between mother and calf pairs [10], [11], [12]. Therefore, we might expect to record more signature whistle types in larger groups or groups where calves are present [11], [22]. We investigated the relationship between the number of different signature whistle types recorded, i.e. the diversity of different types and group size, as well as the influence of calf presence using a generalised additive model (GAM). GAMs were fitted in R (Ver. 2.14) [51] using the ‘mgcv’ package [52], applying a Gaussian distribution and identity link function. The response variable used was the number of unique signature whistle types recorded per encounter. The explanatory variable of group size from field estimates was included as a smoothed term, fitted using a penalised regression spline framework and generalised cross-validation [52]. The presence of calves in the group was included as a binary factor. As the number of whistles can only increase with recording time, we also included recording duration per encounter in minutes in the models as a smoothed explanatory variable and also investigated a potential interaction of this variable with group size. Models were compared using an ANOVA [53].

### Ethics statement

Ethics clearance for this study was granted by the University of Pretoria's Animal Use and Care Committee under permit number EC061-09 to SHE. Research in Namibia was conducted with permission from the Namibian Ministry of Fisheries and Marine Resources (no specific permit required). Acoustic data are held by the Namibian Dolphin Project and can be made available for use on request.

## Results

**Figure 1:**
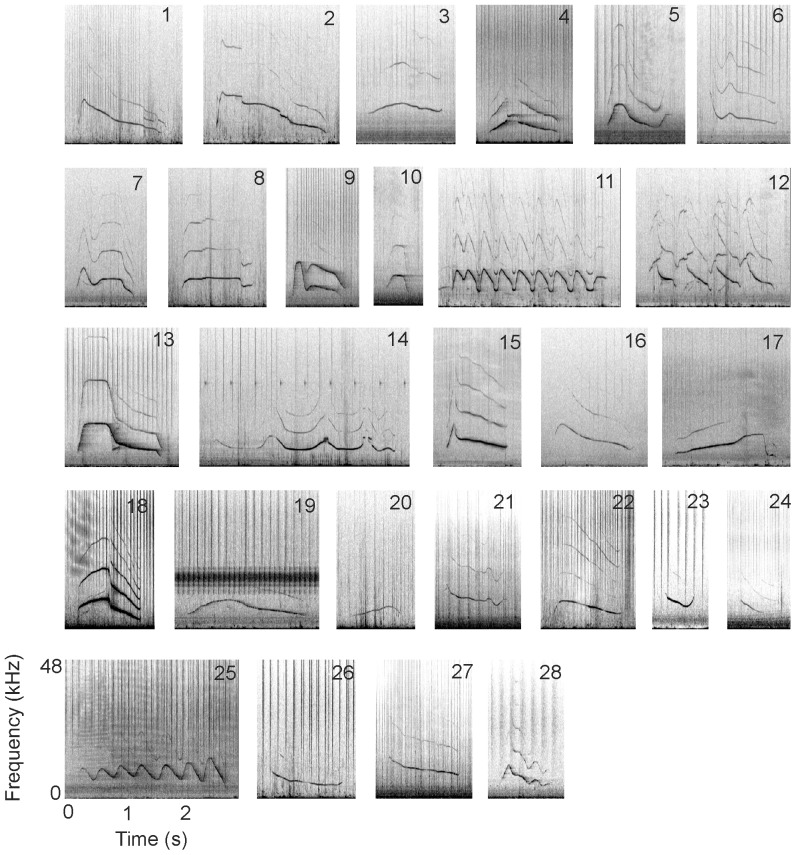
Example spectrograms of all signature whistle types identified (n = 28) from common bottlenose dolphins in Namibia. Frequency (kHz) is on the y-axis and ranges from 0 to 48 kHz. Time (s) is on the x-axis. The scaling is the same for all spectrograms. Spectrogram settings: FFT 512, Hanning window, overlap 50%. The numbers in the top right corner of each spectrogram are the unique SW identification numbers of each signature whistle type.

Recordings were made during 64 encounters over 57 fieldwork days in 2009 and between 2011 and 2013. More than 79 hrs of acoustic data were collected over a range of behavioural contexts, group sizes and compositions. Recording time per encounter ranged from 8 to 208 min, with most encounters (67%) having more than 50 min of acoustic recording effort. Forty-three REWTs were identified and catalogued, of which 28 were subsequently identified as signature whistle types (Figure 1). A 29^th^ signature whistle type was identified but removed after careful consideration because most emissions were low quality (SNR 1 and masked) making stereotypy hard to assess. In most cases (82%), the same signature whistle type was recorded in 2 or more years, with 4 signature whistle types (14%) identified at both Walvis Bay and Lüderitz field sites.

A total of 3770 contours were identified from the entire acoustic dataset. During the classification phase, 918 contours were classified as REWTs, 403 as variable contours and a further 2424 were left unclassified. A high proportion (80%) of low SNR contours were included in the unclassified bin, but in 276 cases (12%) these quality 1 contours were assigned to a REWT category as the entire contour could be determined and classified. Instances of overlapping whistle copying were rare, identified on just 25 occasions. Following classification and bout analysis (SIGID) [23], 820 whistles were classified into one of the 28 signature whistle types. The number of signature whistles per type ranged from 6 to 81 (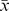
29±SD 20).

Signature whistles were identified from 59% (*n* = 38) of encounters. The vast majority of signature whistle types (93%) were produced during more than one encounter. However multiple encounters of bottlenose dolphins per day were rare, and on no occasion was the same signature whistle type recorded in different encounters on the same day. Descriptive statistics for the frequency of signature whistle type occurrence are therefore the same for encounter and day (Table 2). In addition to their production in a bout, the majority of signature whistle types identified (79%) were produced as single occurrences on one or more days (Table 2). Overall there were 140 occasions where signature whistles belonging to one of the 28 signature whistle types were identified, occurring as either a single whistle (*n* = 42) or in bouts (*n* = 98) containing repetitions of 2 or more signatures produced with an IWI of 1 to 10 s.

**Table 2:** Data summary showing the frequency of occurrence of each signature whistle type and the whistle loop structure.

		Frequency of occurrence			Loop structure (% of category size)		
ID	Whistle category size (single occurrences)	*Encounters / days	Years	Location	SE	CML	DCML
SW 1	33 (3)	5	2011/12/13	WB	85	9	6
SW 2	37 (1)	2	2012	WB	100	0	0
SW 3	15 (1)	4	2011/12	WB	100	0	0
SW 4	10 (0)	3	2012/13	WB	100	0	0
SW 5	49 (2)	8	2011/12	WB	92	2	6
SW 6	37 (1)	3	2012	WB	100	0	0
SW 7	77 (1)	16	2011/12/13	WB/LÜD	99	1	0
SW 8	25 (0)	3	2011/12	WB	72	16	12
SW 9	20 (1)	2	2011/12	WB	95	5	0
SW 10	24 (2)	5	2011/12	WB	67	29	4
SW 11	24 (0)	3	2011/12	WB	4	96	0
SW 12	81 (2)	7	2011/12/13	WB/LÜD	52	47	1
SW 13	51 (4)	13	2011/12/13	WB	98	2	0
SW 14	29 (2)	7	2009/11/12	WB	42	55	3
SW 15	21 (3)	6	2011/12	WB	91	0	9
SW 16	22 (2)	4	2011/12/13	WB	100	0	0
SW 17	35 (1)	3	2012/13	WB/LÜD	100	0	0
SW 18	39 (2)	6	2011/12/13	WB	59	36	5
SW 19	16 (2)	3	2011/12	WB	100	0	0
SW 20	7 (1)	2	2009/11	WB	72	14	14
SW 21	8 (1)	3	2011	WB	100	0	0
SW 22	60 (4)	9	2011/12/13	WB	80	20	0
SW 23	40 (3)	8	2011/12/13	WB/LÜD	100	0	0
SW 24	6 (1)	2	2012/13	WB	100	0	0
SW 25	17 (2)	2	2011/12/13	WB	0	100	0
SW 26	9 (0)	1	2012	LÜD	100	0	0
SW 27	14 (0)	1	2011	WB	100	0	0
SW 28	14 (0)	5	2009/11/13	WB	100	0	0
Mean ±SD	29±20	5±4	2	n/a	81	17	2

*As there was no difference in the data for the occurrence of signature whistle types during encounters or days, this has been displayed as one column.

The number of encounters/days when signature whistles were identified as single occurrences is shown in parenthesis next to category size. Location: WB =  Walvis Bay, LÜD =  Lüderitz. Whistle structures are SE =  single element, CML =  connected multi-loop, DCML =  disconnected multi-loop.

The diversity of signature whistle types, i.e. the number of different signature whistle types identified, varied considerably between encounters. When present, the mean number of different signature whistle types per encounter was 3.68, with a maximum of 14 different types identified from a single encounter. However, signature whistle type diversity per encounter was usually low: 61% of the encounters containing signature whistles had between 1 and 3 different signature whistle types and 39% had more than 3 different types (4 to 14).

The visual classification task tested the reliability of identifying whistle types. The first stage, a pair-wise comparison involving a similarity rating of 50 whistle repeats with each of 10 templates, indicated inter-observer agreement was low (Fleiss' kappa statistic without author as judge: κ = 0.216, *n* judges  = 5, *Z* = 25.9, *P*<0.001; with author as judge κ = 0.238, *n* judges  = 6, *Z* = 35.3, *P*<0.001). When judges were forced to make a binary decision on matching whistle repeats to templates, they repeatedly (99.60% of times) chose a whistle with their highest or joint highest similarity rating from phase 1 as the most similar whistle to the template category. Classification agreement amongst judges in stage 2 was high (Fleiss' kappa statistic without author as judge: κ = 0.848, *n* judges  = 5, *Z* = 55.3, *P*<0.001) and in 89% of cases matched that of the author. Most disagreement in classification between the author and judges (64% of classifications that differed) was caused by ambiguity in one whistle type. Overall, these results demonstrate that clearly defined whistle types exist in the repertoire of Walvis Bay bottlenose dolphins and lend support to the authors' visual categorisation of the dataset.

**Figure 2:**
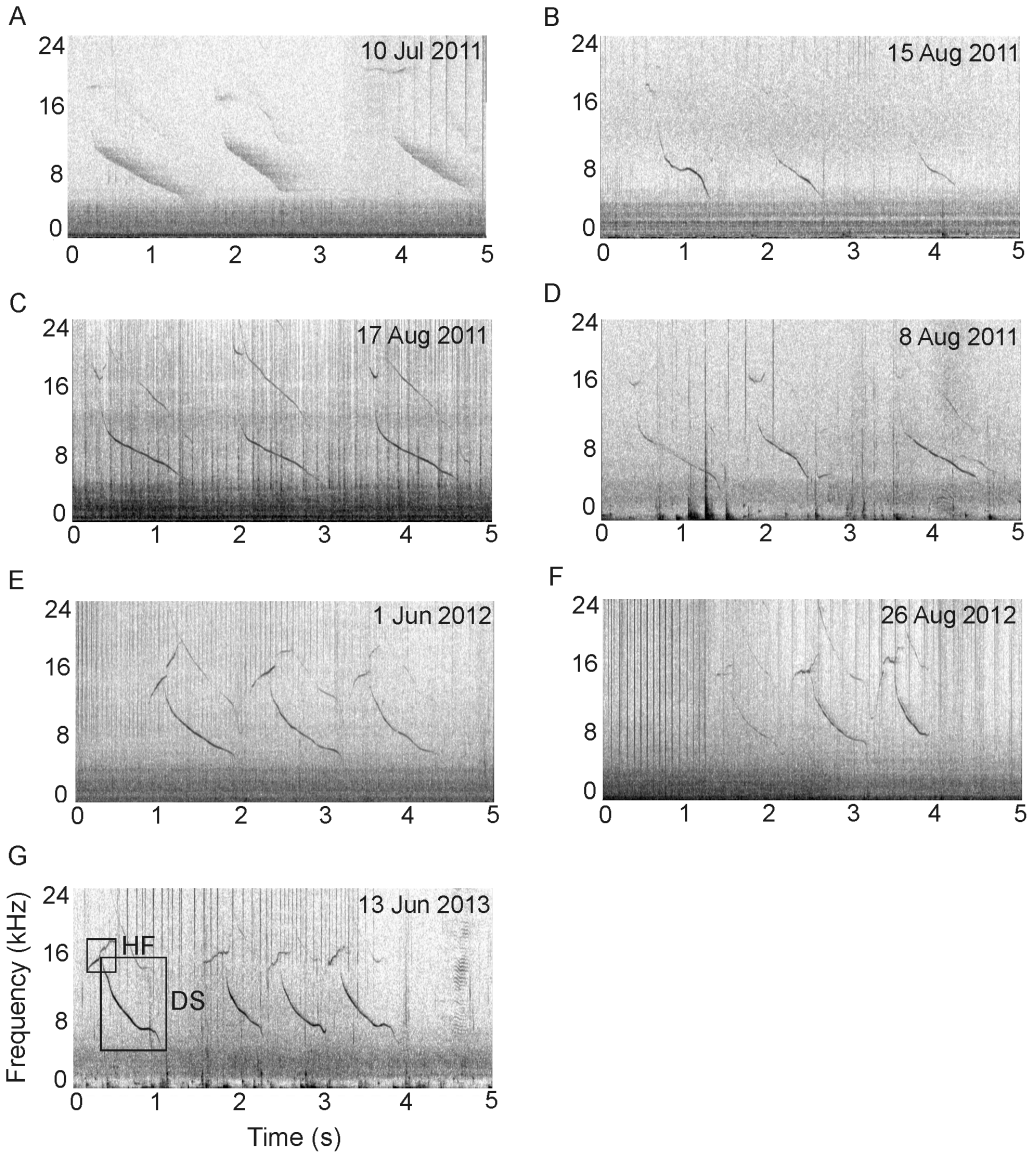
Spectrograms of signature whistle SW 12 showing possible two-voice whistle production by a common bottlenose dolphin. SW 12 was recorded in 3 different years during 7 encounters/days (2011: A–D, 2012: E–F, 2013: G). Frequency (kHz) is on the y-axis and ranges from 0 to 24 kHz. Time (s) is on the x-axis. The scaling is the same for all spectrograms. Spectrogram settings: FFT 512, Hanning window, overlap 50%. Note reverberation of whistle in spectrogram A. Loops are highlighted by boxes in spectrogram G: HF =  High frequency contour, DS =  Down-sweep contour.

Walvis Bay bottlenose dolphins predominantly produced single element signature whistles with almost half (13 out of 28) of the signature whistle types identified only ever produced as a single element. Only one signature whistle type (SW 25) was always produced as a connected multi-loop, whereas several more were predominantly single element but occasionally produced as connected or disconnected multi-loops (Table 2). Just one signature whistle type with a non-identical disconnected multi-loop structure was identified (SW 12) and this was unusual in that on almost all occasions (*n* = 143, 94%) where the 2 occurred together, the initial loop (a high frequency contour) overlapped the second (a down sweeping contour) by 0.02 to 0.27 s (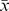
0.12 s±SD 0.06) and often this 2 contour structure was repeated to form a connected or disconnected multi-loop whistle (Figure 2). The initial loop (high frequency), lasting on average 0.27 s (±SD 0.10 s), was never found in isolation, whereas on 9 occasions (6%) the second loop (down-sweep) did appear in isolation. Of these instances, 6 were low SNR whistles (quality 1) and 3 were intermediate SNR (quality 2). Signature whistle type SW 12 was identified on 7 recording days, with a minimum of 3 repeats with overlapping high frequency and down-swept loops on each occasion. It is unusual to have a signature whistle type composed of 2 loops overlapping in time. However, the number of days both contours were recorded together, their consistent timing and the similar relative amplitude of the high frequency and down-swept contour, lend support for their categorisation together as a unique signature whistle type.

Of the 28 signature whistle types identified, 20 were produced in sequences with 4 or more high quality whistles produced consecutively. Table 3 provides a summary of the parameter means (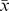), the standard deviation (SD) and coefficient of variation (CV = SD/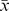
×100) for each of these 20 signature whistle types that were included in the parameter analysis. The mean minimum frequencies ranged from 2.59 to 8.29 kHz and the mean maximum frequencies ranged from 9.88 to 20.06 kHz. Signature whistle SW 12 was the highest frequency signature whistle type measured, with one whistle reaching 22.58 kHz. Mean peak frequencies for all signature whistle types measured were lower than 11 kHz (population 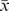
8.20 kHz±SD 1.27) and varied little between whistle types (CV 15.52). On average, the start frequencies (population 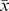
8.02 kHz±SD 3.42) and end frequencies (population
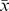
6.42 kHz±SD 2.28) of signature whistle types were similar. However, these parameters were highly variable between signature whistle types (CV 42.62 and 35.48 respectively), probably reflecting inter-individual distinctiveness in whistle types.

**Table 3:** Mean ±SD (CV) acoustic parameters of signature whistle types (*n* = 20) from Walvis Bay bottlenose dolphins.

		Frequency (kHz)						Shape			
ID	*n*	Start	End	Minimum	Maximum	Range	Peak	Start aspect	End aspect	Duration (s)	Inflection (*n*)
SW 1	10	8.17±1.70 (20.82)	4.52±2.18 (48.24)	4.22±2.15 (50.81)	15.39±0.74 (4.82)	11.16±2.30 (20.64)	7.16±3.27 (45.67)	1.00±0.00 (0.00)	−1.00±0.00 (0.00)	1.55±0.56 (45.67)	1.00±0.00 (0.00)
SW 2	10	6.64±0.60 (9.08)	4.76±2.10 (44.15)	4.43±1.21 (27.38)	18.42±1.00 (5.15)	13.99±1.47 (10.53)	9.21±3.22 (34.93)	1.00±0.00 (0.00)	−0.80±0.63 (0.79)	1.83±0.24 (13.35)	1.00±0.00 (0.00)
SW 5	10	6.24±1.21 (19.46)	8.58±1.93 (22.56)	5.66±0.48 (8.44)	13.42±0.60 (4.44)	7.76±0.60 (7.73)	8.93±1.56 (17.52)	1.00±0.00 (0.00)	−1.00±0.00 (0.00)	1.11±0.18 (16.66)	1.20±0.40 (33.33)
SW 6	10	16.51±1.28 (7.75)	6.73±0.50 (7.42)	6.55±0.25 (3.84)	16.51±1.28 (7.75)	9.96±1.30 (13.05)	8.96±1.81 (20.04)	1.00±0.00 (0.00)	−1.00±0.00 (0.00)	1.10±0.07 (6.46)	2.00±0.00 (0.00)
SW 7	6	5.48±2.48 (45.32)	8.00±1.77 (22.16)	4.76±1.59 (33.37)	12.07±0.87 (7.20)	7.32±1.20 (16.38)	8.16±3.34 (40.99)	1.00±0.00 (0.00)	−0.14±0.38 (2.71)	2.12±0.81 (38.08)	4.00±1.15 (28.87)
SW 8	10	6.22±0.70 (10.75)	6.32±1.51 (23.94)	5.52±1.02 (18.50)	10.27±0.18 (1.74)	4.75±1.15 (24.13)	8.12±1.63 (20.11)	1.00±0.00 (0.00)	−1.00±0.00 (0.00)	1.29±0.44 (33.79)	1.30±0.64 (49.25)
SW 9	9	3.90±1.02 (26.19)	5.77±1.15 (20.01)	3.9±1.02 (26.19)	15.68±1.42 (9.05)	11.78±1.65 (13.99)	7.75±2.75 (29.36)	1.00±0.00 (0.00)	−1.00±0.00 (0.00)	0.60±0.28 (45.87)	2.33±0.94 (40.41)
SW 10	10	6.45±0.78 (12.05)	7.22±1.14 (15.77)	6.16±0.85 (13.84)	11.66±0.74 (6.33)	5.50±0.63 (11.41)	10.67±1.06 (9.89)	1.00±0.00 (0.00)	−1.00±0.00 (0.00)	0.47±0.04 (9.05)	1.00±0.00 (0.00)
SW 11	7	5.28±1.84 (34.82)	9.30±0.63 (6.82)	4.42±0.45 (10.24)	13.28±0.35 (2.62)	8.86±0.43 (4.83)	8.92±2.31 (25.94)	−1.00±0.00 (0.00)	−1.00±0.00 (0.00)	2.35±0.82 (35.72)	12.71±4.03 (31.66)
SW 12	10	13.3±1.28 (9.60)	6.12±2.34 (38.15)	5.43±0.53 (9.71)	20.06±1.41 (6.97)	14.63±1.76 (12.03)	9.28±1.24 (13.38)	1.00±0.00 (0.00)	−0.40±0.97 (243)	1.51±0.75 (49.71)	4.00±2.57 (64.23)
SW 13	6	6.41±1.48 (23.12)	2.76±1.00 (36.09)	2.76±1.00 (36.09)	15.19±0.60 (4.42)	12.43±1.42 (11.46)	5.91±1.94 (32.85)	0.50±0.93 (186)	−1.00±0.00 (0.00)	1.47±0.19 (13.11)	1.00±0.00 (0.00)
SW 14	6	9.33±1.70 (18.62)	6.07±0.89 (14.56)	5.48±0.11 (1.96)	9.88±1.10 (10.60)	4.40±1.11 (25.29)	6.78±1.21 (17.78)	1.00±0.00 (0.00)	−1.00±0.00 (0.00)	2.03±0.31 (15.12)	4.33±0.75 (17.2)
SW 15	9	10.51±3.04 (28.94)	7.56±2.00 (26.45)	6.43±0.79 (12.21)	14.38±2.26 (15.70)	7.95±1.95 (25.01)	8.67±1.92 (22.11)	1.00±0.00 (0.00)	−1.00±0.00 (0.00)	1.12±0.16 (14.48)	2.22±1.23 (55.23)
SW 16	6	9.64±4.75 (49.28)	6.53±0.73 (11.21)	4.98±0.89 (17.86)	14.64±0.51 (3.49)	9.66±1.24 (12.80)	8.31±1.60 (19.28)	0.33±1.03 (321)	−1.00±0.00 (0.00)	1.58±0.09 (5.84)	2.67±4.59 (172.12)
SW 17	10	4.78±0.27 (5.60)	2.59±0.49 (19.13)	2.59±0.49 (19.13)	11.66±0.20 (1.80)	9.08±0.46 (5.02)	7.05±1.14 (16.11)	1.00±0.00 (5.15)	−0.20±1.03 (515)	1.81±0.06 (3.12)	1.00±0.00 (0.00)
SW 18	10	7.24±0.55 (7.62)	3.87±1.74 (45.00)	3.64±1.33 (36.58)	11.07±0.90 (8.09)	7.43±1.80 (24.20)	6.13±0.92 (14.99)	1.00±0.00 (0.00)	1.00±0.00 (0.00)	1.82±0.73 (40.11)	3.80±3.12 (82.21)
SW 19	10	5.35±0.91 (16.92)	4.95±0.36 (7.33)	4.80±0.54 (11.25)	10.41±0.26 (2.50)	5.61±0.69 (12.35)	6.99±0.96 (13.79)	−1.00±0.00 (0.00)	−1.00±0.00 (0.00)	2.15±0.10 (4.62)	1.00±0.00 (0.00)
SW 22	8	4.41±1.00 (22.69)	6.26±2.04 (32.52)	3.56±1.33 (37.34)	10.20±0.50 (4.86)	6.64±1.47 (22.17)	7.52±0.94 (12.46)	1.00±0.00 (0.00)	0.00±1.04 (0.00)	1.34±0.22 (16.19)	1.63±0.52 (31.85)
SW 23	9	11.89±1.74 (14.63)	12.24±1.22 (9.97)	7.59±0.38 (5.01)	12.79±1.26 (9.87)	5.21±1.19 (22.93)	9.63±1.43 (14.87)	1.00±0.00 (0.00)	−1.00±0.00 (0.00)	0.46±0.06 (12.42)	1.00±0.00 (0.00)
SW 24	10	12.59±4.02 (31.91)	8.29±0.65 (7.83)	8.29±0.65 (7.83)	13.91±0.97 (6.97)	5.62±0.95 (16.84)	9.79±1.06 (10.87)	1.00±0.00 (0.00)	0.17±0.75 (441)	1.12±0.14 (12.75)	0.00±0.00 (0.00)
Mean±SD (CV)	9	8.02±3.42 (42.62)	6.42±2.28 (35.48)	5.06±1.48 (29.32)	13.54±2.80 (20.67)	8.49±3.07 (36.19)	8.20±1.27 (15.52)	0.77±0.62 (80.52)	−0.67±0.56 (83.58)	1.44±0.55 (38.20)	1.72±1.34 (77.93)

Eleven of the 20 signature whistle types measured had little (<3 kHz) difference in mean start and end frequency, of which 9 were produced with a similar overall shape, having a non-looped (single element) structure, beginning with a positive aspect, ending with a negative aspect and having just 1 inflection point (Table 3). There was greater variation in the overall shape and duration of the remaining signature whistle types measured. For example, signature whistle type SW 11, a connected multi-loop, was produced with 7 to 20 inflection points and consequently the average duration of this signature whistle type was relatively long (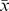
2.35 s±SD 0.82) and variable (within signature whistle type CV of 35.72).

As DCML signature whistles were rare, assessments of loop temporal production were limited by a small sample size (*n* = 15 intervals measured). For whistles produced as disconnected multi-loops, the mean ILIs ranged from 0.09 to 0.18 s with a population average of 0.14 s (±SD 0.03 s). The population mean IWI was 9.32 s (±SD 7.67), and most (64%) were produced with mean IWIs of under 10 s and the median IWI was 6.76 s. The longest IWI was 184.53 s for signature whistle type SW 2. Estimates of IWI are likely to be conservative due to the occurrence of boat noise intermittently during recordings which reduced the likelihood of identifying long IWIs.

**Figure 3:**
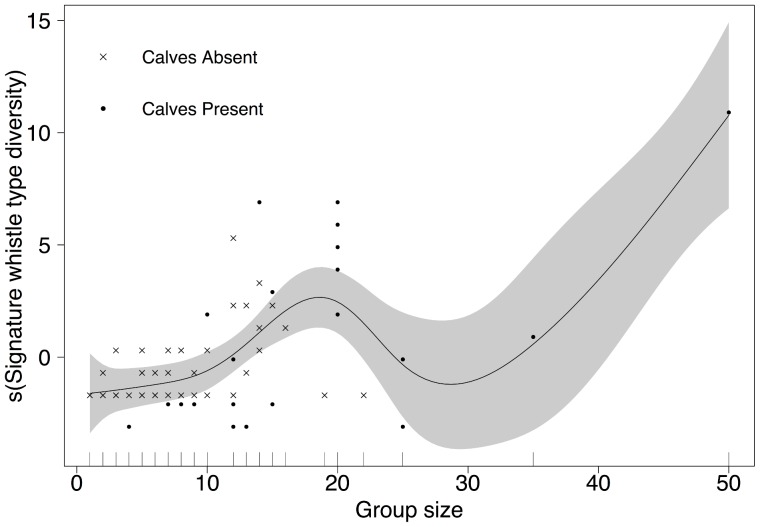
GAM response curve showing the effect of group size on the diversity of signature whistle types of common bottlenose dolphins from Namibia. Generalized additive model response curve showing smoothed fit of the relationship between signature whistle type diversity and group size per encounter, controlled for calf presence. The plot controls for the relationship of other variables, and is a result of back-fitting the algorithm used by the R-function GAM to calculate the additive contribution of each variable using nonparametric smoothing methods. Note that the y-axis is on the scale of the link function, not the measured variable. Points represent the residuals, the solid line represents the function estimated by the GAM and the area in grey shows the 95% confidence interval.

Group size and calf presence had a significant effect on the diversity of signature whistle types recorded per encounter (Table 4). The best fitting model, which included group size as a smoothed parameter and calf presence as a binary variable, explained 59.2% of the deviance. Although inclusion of recording duration fractionally increased the amount of deviance explained to 59.9% it did not significantly improve model fit (ANOVA: *F* = 1.16, *P* = 0.275). The diversity of signature whistle types increased with group size, with an almost linear relationship in groups of 1 to 20 individuals (Figure 3). Interpretation of model results for larger groups is hampered by the low number of data points from groups of more than 20 individuals. On average there were 4.2 different signature whistle types detected in groups containing calves (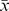
group size when calves present  = 17.42), compared to 1.2 signature whistle types in groups without calves (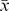
group size when calves absent  = 8.19).

**Table 4:** Summary of GAM output investigating the diversity of signature whistle types of common bottlenose dolphins from Namibia.

Model	Covariates	AIC	GCV Score	% Deviance explained
1	group size	293.567	5.578	42.6
2	s(group size)	286.304	5.033	55.7
3	calf presence +s(group size)	283.223	4.817	59.2
4	calf presence +s(group size) +s(recording mins)	283.795	4.879	59.9
5	calf presence +s(group size) +s(group size × recording mins)	283.795	4.879	59.9
6	calf presence +s(group size) +s(group size × calf presence)	283.227	4.683	53.1

Details of the 6 models including the measures of goodness of fit - the generalized cross -validation (GCV) and Akaike's Information criterion (AIC) scores - used during selection of models to predict signature whistle type diversity in common bottlenose dolphin encounters in Namibia. The interaction term in model 5 and 6 did not improve model fit and the 3 remaining best fitting models were compared in a pair-wise fashion (2 to 3, 3 to 4, 2 to 4) using ANOVA to investigate the relative contribution of additional terms. Covariates were entered as factors or smoothed terms (indicated by ‘s’), interaction between terms is indicated by ‘x’.

## Discussion

As signature whistles encode individual identity information [6], their characteristics may differ systematically from those of other whistle types in the repertoire of bottlenose dolphins. Identification and characterisation of these calls from wild unrestrained animals is a necessary pre-requisite to understand how they are used in natural circumstances and whether utilisation varies between populations. In this study we identified 28 signature whistle types from wild common bottlenose dolphins residing in Walvis Bay, Namibia. This is the first record of signature whistle use in any population of common bottlenose dolphins inhabiting African waters.

Once developed, signature whistle types may remain stable for decades [9], [19] and possibly throughout life. Stable call structure is necessary for long term call recognition and recent evidence suggests that, like humans [54] and probably elephants [55], dolphins are capable of long term social recognition, recognising signature whistles for up to 20 years without contact between the animals [56]. Although limited to a 5 year time span, our results indicate stability in the signature whistle types of bottlenose dolphins from Walvis Bay with most (82%) recorded on 2 or more years, and 4 identified at Walvis Bay and a second field site, Lüderitz, approximately 450 km away.

Vocal production learning, whereby signal structure is modified as a result of auditory experience [57], plays an important role in signature whistle type development, helping to generate a distinct individualised whistle contour with a unique frequency modulation pattern [5]. The relatively low agreement from the first stage of the task suggests that the frequency modulation pattern of whistle types from Walvis Bay dolphins are not particularly distinct. Common features like the start and end aspect of slope (Figure 1, Table 3) may have led judges to rate whistles as similar As calves use auditory information from their environment during whistle development [16], [17], a shared acoustic environment and socially mediated vocal learning may reduce the distinctiveness of signature whistle types observed within a population compared to that observed between populations. This effect may be more noticeable in small populations such as Walvis Bay, where the number of acoustic models on which calves can model their signature whistle type is limited by the small population size.

Apart from socially mediated learning there are other environmental, ecological, behavioural, genetic and morphological factors that may influence signature whistle characteristics. For example, as animals share a physical habitat, independent acoustic adaptation to promote signal transmission [58] may lead individuals to develop a whistle type with a similar modulation pattern or acoustic parameters, if these propagate particularly well in a given environment. Signature whistle acoustic parameters may also vary with behavioural context [59] and the temporal production of whistles or loops can be affected by stress [29]. Determining the relative contribution of different influences on signature whistle variation is difficult to achieve with a single population and best approached from examination of multiple individuals and populations using recordings collected across a range of behavioural contexts.

Although some signature whistle types used by dolphins in Walvis Bay share common features in the whistle frequency modulation pattern, this does not does not preclude encoding of signature information [21], [60]. The second stage of the classification task was binary, with the judges asked to assign each whistle to a single signature whistle type category. The results of stage 2 demonstrated high agreement between judges, showing that whistle types could reliably be identified in the Walvis Bay data set by multiple human visual judges. As dolphins can discriminate between whistle types which are perceived as similar by humans [61], the subtle but consistent differences in whistle frequency modulation identified by human judges are likely to be perceived as different signature whistle types by bottlenose dolphins in Walvis Bay.

Signature whistles have been identified from wild common bottlenose dolphins in both Sarasota Bay, e.g. [29], [62] and East Scotland [12], [14], as well as in other *Tursiops* species [24]. However, as the acoustic parameters of signature whistles from East Scotland have not been described in detail, Walvis Bay is only the second wild population of common bottlenose dolphins from which these data are available. In general, the acoustic parameters of signature whistle types of common bottlenose dolphins from Walvis Bay are within the range of those reported from other studies of signature whistles from Sarasota Bay and captivity [16], [28], [30], [38], [61], [62]. Most notably, the average duration of whistles was strikingly similar to those reported by Esch *et al.*, [28] for Sarasota Bay (mean ranges of 0.46 to 2.35 s for Walvis Bay and 0.5 to 2.3 s for Sarasota Bay). The frequency parameters of signature whistles in Sarasota Bay and Walvis Bay were similar, although whistles range higher in Sarasota Bay. For example, mean minimum frequencies range from 3 to 13.3 kHz in Sarasota Bay compared to 2.59 to 8.29 kHz in Walvis Bay and mean maximum frequencies range from 9.3 to 27.3 kHz in Sarasota Bay compared to 9.88 to 20.06 kHz in Walvis Bay [28]. Although based on signature whistles recorded in Sarasota Bay during brief capture-release events, subsequent comparisons found no difference in the minimum or maximum frequency of signature whistles recorded under capture-release and undisturbed conditions [29] and Buckstaff [62] quotes a similar frequency range of 2.91 to 23.48 kHz for the same Sarasota Bay population.

Walvis Bay bottlenose dolphins produce mainly single element signature whistles, with only 2% of all signature whistles identified produced in a disconnected multi-loop form. Therefore, although the mean ILI of 0.14 s for Walvis Bay was greater than the mean ILI of 0.10 s reported by Esch *et al.*, [28] from temporarily restrained dolphins in Sarasota Bay, our results are likely to be confounded by small sample sizes. The predominant use of single element rather than looped signature whistles in Walvis Bay and the wide spacing of loops, as suggested by the limited ILI data, could be an adaptation to reduce the effect of reverberation on signal degradation (see Figure 2A for example of reverberation). A similar phenomenon has been shown in birds inhabiting closed habitats such as densely vegetated forests [63], [64]. Forward masking caused by reverberation accounted for a high proportion of the ‘unclassified’ contours that were otherwise of good SNR (quality 2 and 3).

In describing the signature whistles of this wild population, we found evidence of two-voiced (bi-phonation) whistle production. Signature whistle type SW 12 was identified 81 times on 7 different occasions and in both Walvis Bay and Lüderitz field sites. In the majority of cases, this whistle consisted of 2 elements, a high frequency and down-sweep contour, which overlapped by 0.02 to 0.27 s. The higher frequency element was not a harmonic or sideband of the first (Figure 2), and the overlapping duration was too structured to be explained as an artefact of reverberation. Although the occurrence of 2 individuals exchanging overlapping calls back and forth cannot be discounted [12], it seems unlikely that this would have occurred in such a consistent way, with the same contours involved on 7 different recording occasions across the two field sites.

Bi-phonation appears common in the discrete pulsed calls of killer whales [65] and may occur in almost 10% of beluga whale vocalisations [66]. Simultaneous emission of clicks and tonal calls has been described in bottlenose dolphins [67]; however reports of two-voice whistle production are lacking. If the sound production mechanism for whistles and clicks is similar [68], [69], and if both sets of phonic lips can produce both classes of call [70], then two-voice whistle production may be possible and could explain the structure of signature whistle type SW12. Signal complexity may be increased by using two-voice sound production, which can enhance signal identity coding [71] and could convey additional directional information [65], [72]. These features may be beneficial for signature whistles. Even so, two-voice whistle production appears rare in bottlenose dolphins, perhaps in part because it is difficult to identify in wild populations. Using a criteria of similar sound amplitude and simultaneous occurrence of 2 calls can provide evidence of this in wild populations [66]. However, as sound production in odontocetes is a complex phenomenon, a controlled experimental set up, e.g. [70], is required to conclusively demonstrate two-voice whistle production in bottlenose dolphins.

When devising the SIGID method, Janik *et al.*, [23] tuned it to be conservative so that false positives were eliminated. The result was that for the 2 study populations it was tested on (Sarasota Bay and a captive colony) only approximately 50% of signature whistle types were correctly identified. By first identifying REWTs and creating a REWT catalogue, we were able to assess what effect the 1 to 10 s IWI criteria applied in SIGID had on the identification of signature whistle types. All REWTs with 4 or more whistles contained at least one bout which meant they qualified as a signature whistle type (i.e. 4 or more whistles of the same type occurring in a bout where 75% or more are produced within 1 to 10 s). Using these criteria, 29 signature whistle types were identified, of which one was later discounted. Matching whistles to the REWT catalogue also allowed us to determine whether signature whistles were produced in sequences with IWIs greater than 10 s. When occurring in bouts, the mean IWI of most signature whistle types was under 10 s, but 36% of signature whistle types had mean IWIs greater than the 10 s cut off applied in SIGID. These values most likely underestimate the mean IWI per signature whistle type because a) the 10 s cut off was initially used to create the REWT catalogue, so that signature whistle types which are only ever produced at IWIs greater than 10 s would have been missed, and b) boat noise occurred intermittently in our recordings and would therefore reduce the likelihood of identifying long IWIs between repeated whistles of the same type. These results indicate that although the SIGID method worked well for Walvis Bay and is a promising method for other wild populations, users of this approach cannot guarantee identification of all signature whistle types within the population.

Signals which encode vocal identity can be useful conservation tools [73] and it may be possible to use signature whistles in a mark-recapture framework to assess individual habitat use, ranging patterns or population size [23]. Linking signature whistle types to individuals who are freely interacting is difficult when using a single hydrophone approach and stereo acoustic tags [74] or acoustic localisation [9], [12] are better suited for this. However, the Walvis Bay population is relatively small and by comparing photo-identification data with signature whistle data per encounter, it may be possible to narrow down which signature whistle type belongs to which catalogued animal, by a process of elimination [22]. For example, signature whistle type SW 28 was recorded during 3 encounters in 2009/11. As only one animal (individual catalogue number T-071) was present in all these encounters the evidence suggests that SW 28 belongs to dolphin ID T-071. Providing there are sufficient encounter data linking signature whistles to individuals in this way, a passive acoustic monitoring programme which uses signature whistles to investigate individual habitat use could be implemented. In the long run this may be more cost effective then photo-identification surveys.

However, signature whistles were not ubiquitous in our recordings. Only 58% of encounters (*n* = 37) contained signature whistles, leaving a substantial amount of field effort (*n* = 27 encounters) where no signatures were detected. As signature whistles are cohesion calls, usually produced when animals are out of visual contact [10], it is not surprising that they were not identified in every encounter. The population of bottlenose dolphins in Walvis Bay is small and in most cases only one group of dolphins was encountered per day, reducing the chance for signature whistles to be recorded during exchanges between groups meeting at sea [12]. Signature whistle production also varies with behavioural context, with higher rates during socialising compared to travelling [22]. We recorded dolphins over different behavioural states and group separation. During resting and travelling they were often bunched and thus likely to have been in contact visually or through perception of each other's echolocation clicks [75], [76], thereby reducing the need for signature whistles. We found that signature whistle type diversity per encounter increased with group size and was positively related to calf presence (Figure 3), which suggests that mother and calf pairs use signature whistles to remain in contact [11]. However, due to the small number of data points and the strong relationship between calf presence and group size, it was not possible to tease apart the relative contributions of these variables on the signature whistle type diversity per encounter, other than both appear to have an influence.

Temporal patterning is common in contact calls [77], [78] and can optimise information transfer by increasing redundancy, particularly in noisy habitats [79]. As signature whistles can be identified by their occurrence in bouts [12], [14], [23], there are few data on how often they are produced as single occurrences in the wild, which may be more frequent than currently understood. By classifying whistles to the REWT catalogue we showed that most signature whistle types identified (79%) were produced as single occurrences on at least one occasion. As the ambient noise levels in Walvis Bay are relatively low (authors unpublished data), signal transmission in some circumstances may be effective without call repetition. Alternatively, the function of signature whistles produced in bouts may differ from that of signatures produced as single occurrences, with single occurrences potentially used to seek attention and communicate identity information when animals are in close range or in visual contact.

Signature whistles act as individually specific labels for different social companions [14] and referential signalling using learned signatures may explain signature whistle copying [13], [47], [80], [81]. Through copying, dolphins integrate the signature whistles of others into their own repertoire [80], which may be recalled and produced later when the owner is not present [9]. Therefore, although whistles produced in repeated bouts of the same whistle type, separated by 1 to 10 s, are highly likely to be produced by the same individual [23], those produced as single occurrences could be signature whistle copies [47]. However, the available data suggests that whistle copying occurs rarely [13], [47] and the acoustic parameters of copies differ from the original [13], therefore it is unlikely that copies were matched to a REWT category during visual classification. Furthermore, copies mostly result in a reply by the signature whistle owner [13], [14] and therefore would not be evident as single occurrences. Given the characteristics of copying behaviour, we think that vocal copying of signature whistles is unlikely to have greatly affected our results. It therefore seems likely that individuals produce their signature whistles both in bouts and as single occurrences.

## Conclusions

Using a relatively simple method involving a single hydrophone and bout analysis [23] we have identified and described 28 signature whistle types from a wild population of common bottlenose dolphins inhabiting Walvis Bay. The relative stability and dominance of signature whistles in the repertoire of bottlenose dolphins enables researchers to look more deeply into the cognitive abilities of dolphins [82]. The study of signature whistles therefore helps to answer questions relating to vocal learning [16], individual recognition [6], referential communication [14] and long term memory [56] in non-human animals. Future studies can build on the existing signature whistle catalogue, to help address some of these topics for this population in Walvis Bay.

## Supporting Information

Figure S1 **Visual assessment of SNR: example.** Example of one whistle type (SW 22) with 3 different SNR ratings - 1 to 3. Frequency (kHz) is on the y-axis and ranges from 0 to 48 kHz and time (s) is on the x-axis. The scaling is the same for all three spectrograms. Spectrogram settings: FFT 512, Hanning window, overlap 50%. Note the presence of a second concurrent whistle type in the SNR 2 and (more notably) in the SNR 3 example.
(TIF)
