# Supplementary figures and images for: Identification and Characteristics of Signature Whistles in Wild Bottlenose Dolphins (Tursiops truncatus) from Namibia
Source: PLoS One. 2014 Sep 9;9(9):e106317. doi: 10.1371/journal.pone.0106317 (PMC4159226; doi:10.1371/journal.pone.0106317)

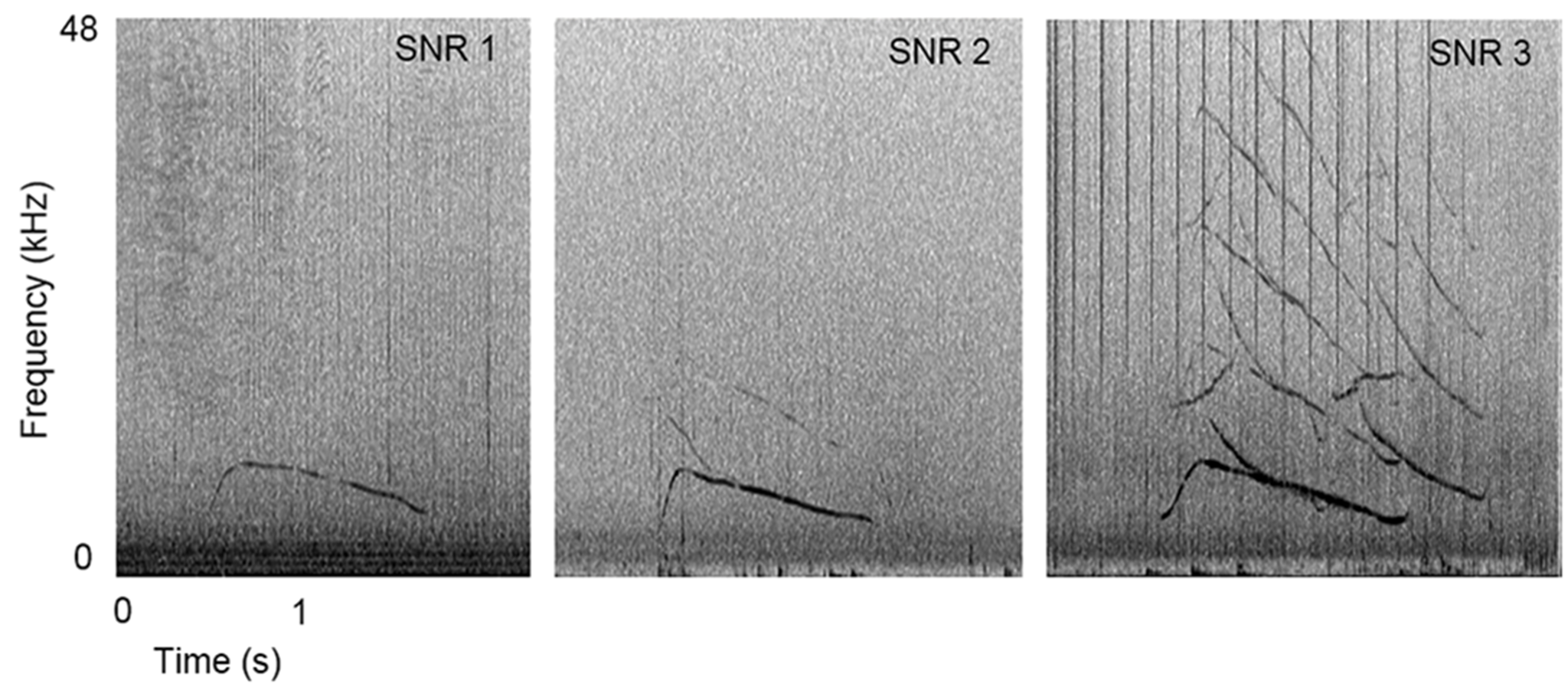

Supplement: Figure S1 — Visual assessment of SNR: example. Example of one whistle type (SW 22) with 3 different SNR ratings - 1 to 3. Frequency (kHz) is on the y-axis and ranges from 0 to 48 kHz and time (s) is on the x-axis. The scaling is the same for all three spectrograms. Spectrogram settings: FFT 512, Hanning window, overlap 50%. Note the presence of a second concurrent whistle type in the SNR 2 and (more notably) in the SNR 3 example. (TIF) [file pone.0106317.s001.tif]
